# Yeast Strain Influences the Hop-Derived Sensory Properties and Volatile Composition of Beer

**DOI:** 10.3390/foods12051064

**Published:** 2023-03-02

**Authors:** Ashly Kumar, Andrea Warburton, Patrick Silcock, Phil J. Bremer, Graham T. Eyres

**Affiliations:** Department of Food Science, University of Otago, P.O. Box 56, Dunedin 9054, New Zealand

**Keywords:** beer fermentation, hops, yeast strains, terpene biotransformation, sorting task

## Abstract

The perception of hop-derived flavour in beer is not well understood, particularly regarding the effect that different yeast strains and fermentation parameters have on perceived hop aroma and the mechanisms responsible for these changes. To evaluate the influence of yeast strain on the sensory properties and volatile composition of beer, a standard wort, late-hopped with New Zealand Motueka hops (5 g·L^−1^), was fermented with one of twelve yeast strains under constant conditions (temperature and yeast inoculation rate). The bottled beers were evaluated using a free sorting sensory methodology, and their volatile organic compounds (VOC) were assessed using gas chromatography mass spectrometry (GC/MS) with headspace solid-phase microextraction (SPME) sampling. Beer fermented with SafLager W-34/70 yeast was associated with a hoppy flavour attribute, whereas WY1272 and OTA79 beers were sulfury, and WY1272 was also metallic. WB06 and WLP730 beers were perceived to be spicy, with WB06 beer also perceived as estery, whereas VIN13 beer was sour, and the WLP001 beer was astringent. Beers fermented using the twelve yeast strains had clearly distinct VOC profiles. Beer made with WLP730, OTA29, SPH, and WB06 yeasts had the highest 4-vinylguaiacol levels, which contributed to their spicy attribute. Beer made with W3470 had high levels of nerol, geraniol, and citronellol, which supported its sensory characterisation as being ‘hoppy’. This research has illustrated the important role that yeast strain has on modulating hop flavour in beer.

## 1. Introduction

The perception of flavour and the quality of beer is dependent on the raw materials used and the variety of reactions the happen during wort production and fermentation. Flavour generation reactions during fermentation vary depending on the nature of the ingredients, including the yeast strain used, and the fermentation conditions [1]. Yeasts are essential in beer production where the yeast strain influences the type of beer produced and its flavour profile due to the metabolites generated during fermentation [2]. Yeasts also alter the perception of hop flavour in beer by biotransforming hop flavour precursors during fermentation, with different yeast strains producing beers with differing concentrations of hop-related volatile organic compounds (VOC) [3,4].

Hop flavour in beer is dependent on complex physical, chemical, and biological changes that happen during wort production and fermentation. Hops (*Humulus lupulus* L.) are responsible for the characteristic bitterness of beer that counteracts the sweetness from malt and conveys drinkability to beer. Volatile compounds derived from hop essential oil are responsible for a variety of aroma attributes, particularly when hops are added late in the boil (late hopping) or added during the fermentation or maturation stages of production (dry hopping). The perception of hop flavour also changes during fermentation due to modification of hop compounds by yeast during fermentation (biotransformation reactions) [5]. These yeast–hop interactions generate a diverse range of hop flavours in beer including resinous, fruity, citrusy, floral, and spicy attributes [6]. Despite a large quantity of research on the composition of hop essential oil, much is unknown about the mechanisms by which hops contribute to beer flavour [7].

Important chemical components in hop essential oils are terpene hydrocarbons (including monoterpenes such as β-myrcene, sesquiterpenes such as α-humulene and β-caryophyllene), oxygenated compounds (alcohols, aldehydes, ketones, and esters, which may be terpene derivatives), and sulfur compounds [8,9]. Terpenoids are a diverse class of compounds derived from isopentenyl pyrophosphate precursors, which generate a range of aroma and flavour characters [10]. The biotransformation of monoterpenes by yeast during fermentation has been reported by King and Dickinson [4], where yeast activity converts monoterpene alcohols such as geraniol and linalool to a range of other terpenoid products via either isomerisation (e.g., converting geraniol to nerol), reduction (e.g., to citronellol), or esterification [4,11]. Biotransformation of hop-derived compounds by different yeast strains has been shown to impact on the VOCs profile in beer [3] and has been speculated to also impact on the perceived flavour of beer, although comprehensive studies have not been reported in the literature.

The free-sort method has been demonstrated to be an effective rapid sensory evaluation technique to describe beer characteristics [12]. Free sorting aims to measure the degree of similarity between samples by sorting the samples into groups according to their similarities and differences. The inclusion of an additional descriptive step enables the sensory characterisation of the samples [13].

The aim of this study was to examine the influence of different yeast strains on the volatile composition and the sensory properties of the resulting beers. A standard wort, late-hopped with New Zealand Motueka hops, was fermented using one of 12 different yeast strains, including a selection of beer and wine yeasts, to obtain a diverse range of sensory characteristics. Sensory analysis was carried out using a free sorting task methodology with a panel of 14 assessors, including trained sensory panelists, volunteers, and brewing professionals. The VOC composition of the beers was assessed using gas chromatography mass spectrometry (GC/MS) with headspace solid-phase microextraction (HS-SPME) sampling.

## 2. Materials and Methods

### 2.1. Brewing and Fermentation

Yeast strains (Table 1) were propagated in sterile (autoclaved) malt medium (350 mL, 10% *w*/*v*) for 20 h at 20 °C. Fermentis SafAle US-05, SafAle WB-06, SafAle BE-256, SafLager W-34/70, and SafLager S-23 yeasts were provided by Fermentis (Lille, France); White Labs California Ale WLP001 (San Diego, CA, USA) was purchased from Homebrew West (Auckland, New Zealand); Anchor VIN13 hybridised wine yeast and Exotics SPH wine yeast (Anchor Oenology) were supplied by Scott Laboratories (Petaluma, CA, USA). OTA29 and White Labs Chardonnay White Wine Yeast WLP730 were propagated from agar slopes from the University of Otago yeast collection according to the protocol described below (Section 2.2). OTA79 (University of Otago yeast collection) and Wyeast American Ale II WY strain1272 (Hood River, OR, USA) were supplied as a slurry by The Emerson’s Brewing Company (Dunedin, New Zealand).

Wort was produced at The Emerson’s Brewing Company with an original gravity of 10°P at an efficiency of 80%. Milled malt (194 kg) was added into the mash vessel and mixed with hot water (582 L) to achieve a strike temperature of 50 °C together with CaCl_2_ (100 g), lactic acid (150 mL), and β-glucanase (100 mL). A temperature programmed mash with rests at 50 °C (15 min) and 67 °C (45 min) was utilised to achieve saccharification before increasing the temperature to 75 °C for mash out. The sweet wort was recirculated in the lauter tun for 15 min for clarification before transfer to the kettle for wort boiling. The pre-boil gravity and pH of the wort sample were determined to be 1.038 and 5.33, respectively.

The wort was boiled for 60 min with antifoam and bittering hops (Simcoe; 580 g) to achieve 20 IBU. Kettle finings (koppafloc; 100 g) and magiFood (100 g) were added after 45 min to reduce haze in the final beer. After 60 min, the boil was stopped, and Motueka hops (6000 g total; dose rate of 5 g·L^−1^) were added for a 5-min steep. The wort was transferred into a whirlpool vessel for 10 min for clarification to remove spent hops and trub. Wort was cooled to 18 °C using a heat exchanger during transfer to a holding tank with in-line oxygenation using O_2_ at 50 L·min^−1^ (pressure = 4 psi). From the final wort volume (1200 L), 12 × 10 L aliquots were added into sterile 12 L fermenters. Each wort (10 L) was inoculated with one of the 12 propagated yeasts to achieve a target pitch rate of 1 × 10^7^ viable cells·mL^−1^. Fermentation took place at 20 °C for 9 days until a constant gravity was achieved in all fermenters. The beer was then matured and clarified at 4 °C for 9 days prior to bottling. Beer samples were bottled into 330-mL brown glass bottles with the addition of 1.5 g sucrose to carbonate the beer to 2.0 volumes CO_2_ by holding the samples in an incubator at 20 °C for 14 days.

### 2.2. Yeast Preparation

Yeast cultures were propagated based on the origin of each yeast strain (Table 1), before calculating the required yeast volumes to the fermenter for each yeast strain to achieve the target pitch rate.

#### 2.2.1. Rehydration from Dry Yeast

To initially determine cell density per gram, 1 g of each dry yeast was rehydrated in filtered water (100 mL) (boiled and cooled to room temperature) and stirred for 10 min using a magnetic stirrer. Cell viability (initial cell count) was estimated to determine the amount of yeast suspension required to obtain 1 × 10^7^ cells·mL^−1^ (for a 10 L ferment). For final pitching, dry yeast (11.5 g) was added to a malt solution (10°P, 1 L), incubated for 24–48 h at 20 °C, and revived 48 h prior to yeast enumeration and pitching (Section 2.2.5).

#### 2.2.2. Propagation of Cultures from Agar Slopes (Otago Culture Collection)

Yeast strains on agar slopes in the Otago culture collection required several propagation steps to obtain sufficient numbers for the target pitch rate. Yeasts recovered from agar slopes were inoculated into duplicate 10-mL autoclaved malt solution (10% *w*/*v*) in 20-mL Universal vials using a sterile loop and incubated for 24 h at 20 °C. These cultures (two x 10-mL volumes) were added to 180 mL of sterile malt medium (10% *w*/*v*) in a 500-mL Schott bottle and propagated for 24–48 h at 20 °C. The resulting yeast suspension was added to 2800 mL of autoclaved malt medium (10% *w*/*v*; 10°P) in a 5-L conical flask. The starter culture was stirred for 24–48 h at 20 °C with a magnetic stir bar, prior to yeast enumeration and pitching (Section 2.2.5).

#### 2.2.3. Commercial Slurry

Fresh liquid yeast slurry (250 mL) was provided by Emerson’s from their yeast propagation tanks. The slurry was added into 1 L (in a 3-L conical flask) of fresh brewery wort at 10°P provided by the brewery and stirred for 24–48 h at 20 °C with a magnetic stir bar, prior to yeast enumeration and pitching (Section 2.2.5).

#### 2.2.4. Commercial Yeast Slurry

A vial of White Labs WLP-001 California Ale^®^ yeast was purchased commercially and propagated by adding the vial to 1 L (in a 3-L conical flask) of fresh brewery wort (10°P, 1 L) and stirring for 24–48 h at 20 °C with a magnetic stir bar prior to yeast enumeration and pitching (Section 2.2.5).

#### 2.2.5. Yeast Pitching

Approximately 24 h prior to pitching, each propagated yeast culture was centrifuged (3000 rpm for 10 min at 20 °C) in 1-L bottles (Nalgene 3120-1000 Centrifuge bottle), the supernatant was discarded, Emerson’s fresh brewery wort (10°P, 200 mL) was added to the slurry, and the suspension was agitated (200 rpm, 60 min) to resuspend the yeast. Cell numbers were estimated, and the volume of yeast slurry required to achieve the target inoculation rate of 1 × 10^7^ viable cells /mL in 10 L wort was calculated.

The number of yeast cells in each starter culture (2.2.1–2.2.4) was estimated using an Oculyze BB 1.0 (Oculyze GmbH, Hochschulring, Germany) with methylene blue (MB) as a stain (1:1 ratio). A microscopic slide (200-µL sample chamber; Gräfelfing, Germany) was prepared and analysed under 400× magnification to calculate cell numbers, budding cell values, and culture viability using the cloud-based platform [14]. Five pictures were taken of the most appropriate dilution, and the percentage cell viability (>90% was obtained) and mean yeast numbers per mL (million cells per mL) were estimated [15].

### 2.3. Temperature of Fermentation

*S. cerevisiae* was the most common (8/12) yeast used in this study (Table 1), along with one *S. cerevisiae* hybrid, two *S. pastorianus* strains, and one *S. bayanus* strain. The *S. cerevisiae* stains typically produce an ale-style beer; *S. pastorianus* and *S. bayanus* primarily produce lager-style beers [16]. The use of different yeast strains posed a question of which temperature should be used for fermentation, as this would likely impact on yeast growth rates, fermentation time, and VOC production [16]. Despite lager yeast strains being typically fermented at 8–15 °C and ale yeast strains at 14–20 °C, it was decided to remove temperature as an experimental variable and carry out all fermentations at 20 °C.

### 2.4. Analysis of Beer Samples Using Free Sorting Sensory Methodology

Prior to study commencement, ethics was approved by the University of Otago Human Ethics Committee (Reference 18/154). A total of 14 panelists completed the free sorting task, with eight from the University of Otago Department of Food Science sensory panel, four brewing professionals/expert beer tasters from Emerson’s Brewery, and two Department of Food Science postgraduate students. The free sorting task was completed over five sessions of 2 h each with two initial training sessions used to familiarise the panelists with the sensory space of the beer samples and the free sorting task itself, followed by three formal evaluation sessions. Session one included a taste identification test using five sample solutions (sucrose, citric acid, caffeine, sodium chloride, and alum) followed by a descriptive test where four beer samples from the twelve experimental samples were evaluated. The beers were presented in pairs, and panelists were asked to comment on the sample’s aroma, appearance, flavour, and mouthfeel, as well as their overall impression of the difference between the two beers. In session two, the sorting task protocol was explained, and a mock sorting task was carried out to familiarise the panelists with the sorting task methodology using six of the twelve beer samples.

The formal evaluation sessions were completed during the remaining three sessions. In each evaluation session, the panelists received a tray of all twelve beer samples presented in a balanced order according to a Williams Latin Square design. Beer samples (40 mL) were served at 10 °C ± 2 °C in 200-mL lidded plastic cups identified with random 3-digit codes. The panelists were instructed to smell and taste the samples in the order presented and sort them into groups based on their similarities of sensory attributes. The panelists were instructed to sort the samples into any number of groups, provided that a minimum of two groups and a maximum of 11 groups were formed. A group could contain up to 11 samples if preferred, and a panelist could choose any criteria (sensory attributes) to sort the samples. Panelists also separately recorded individual descriptions on each sample. Retasting was allowed for confirmation of groupings. Once the samples were sorted into groups, the panelists were asked to record the characteristic sensory attributes of each group. The groups and sensory attributes were recorded using Compusense Cloud (Compusense Inc., Guelph, ON, Canada) on Apple iPads (Apple Inc., Cupertino, CA, USA). Filtered water, plain crackers, and sliced carrots were provided as palate cleansers between samples. Maximum alcohol consumption for a panelist at each evaluation session was equivalent to a maximum of 1.50 standard drinks. Panelists were provided with food after each session.

### 2.5. Analysis of Beer VOC Using Headspace Solid Phase Microextraction and Gas Chromatography Mass Spectrometry

The VOC profiles of the beer samples were measured using gas chromatography mass spectrometry (GC/MS) coupled with headspace solid-phase microextraction (HS-SPME). Aliquots of each beer (8 mL) were combined with 2.5 g analytical grade sodium chloride (NaCl; BDH Laboratory Supplies, England) in 20-mL headspace vials and capped with PTFE-lined silicon septa screw caps. Blank samples were prepared with deionised water (8 mL) and NaCl (2.5 g). Each sample was incubated at 40 °C for 5 min with agitation, followed by SPME extraction for 30 min at 40 °C using a multipurpose sampler (Agilent PAL3 RSI 85 Autosampler; Palo Alto, CA, USA). Analysis was completed with an Agilent 6890 N gas chromatograph connected to an Agilent 5975 VL mass spectrometer (MSD) with triple axis detector (Agilent Technologies, USA). Helium was used as the carrier gas in constant flow mode at a rate of 1.0 mL·min^−1^. Separation of analytes was achieved using a Zebron ZB-Wax column (60 m, 0.32-mm i.d., 0.5-μm film thickness; Phenomenex, Torrance, CA, USA). Samples were desorbed in the inlet at 240 °C for 5 min in the splitless mode. The initial oven temperature was 50 °C for 5 min, then heated at 5 °C.min^−1^ to 210 °C, followed by 10 °C.min^−1^ to 240 °C, and held for 5 min. The MSD was operated in electron impact (EI) ionisation mode at 70 eV with an ion source temperature of 230 °C with a scan range of *m/z* 29–300. Analyses were completed in quadruplicate (samples, *n* = 12; blanks, *n* = 4). To prevent order effects, samples were analysed according to a modified Williams Latin Square design.

### 2.6. Data Analysis

#### 2.6.1. Analysis of Sensory Data

The sorting task data, groups, and sensory attributes were exported from Compusense for all evaluation sessions. Due to the free-sort methodology enabling panelists to use their own vocabulary, textual preprocessing was required. This included correcting spelling, standardising word endings, combining synonyms, and selecting key words [17,18]. Multiple researchers confirmed synonymy of attributes and final selection of key words, with any terms used in less than three groups removed from further analysis. The data were initially analysed using the Factorial Approach for Sorting Task data (FAST) [19], which applies multiple correspondence analysis (MCA) to the group and attributes data and projects the data onto a two-dimensional map of the samples and attributes and evaluates significant associations between the samples and attributes. A contingency table of the attributes was generated (Appendix A, Table A1) and used to evaluate the relationship between the sensory and analytical data with the application of multiple factor analysis for contingency tables (MFACT) [20].

#### 2.6.2. Analysis of GC-MS Data

Exported GC-MS data was analysed using PARADISe (PARAFAC2 based Deconvolution and Identification System), version 3.87 [21,22]. This resulted in a table of relative peak area for each detected compound for all samples. Retention indices (RIs) were determined using cubic spline interpolation [23] after running a C9–C30 saturated alkane standard using the same GC temperature program. VOCs were regarded as “unknown” if the mass-spectra match value was below 700 or the calculated RI did not match the reported RI. The relative peak area table was analysed by one-way analysis of variance (ANOVA) with a level of confidence of 95% (Appendix A, Table A2) followed by Tukey post-hoc testing to identify significant groupings.

#### 2.6.3. Analysis of the Relationship between the Sensory and GC-MS Data

Due to the ability of multiple factor analysis (MFA) to analyse multiple data sets of variables collected from the same set of samples, it is particularly useful when investigating the relationship between different experimental measures [24]. MFA produces sample and attribute projections that represent the similarities between the samples and between the different data sets of variables. The extension of MFA to include contingency tables (MFACT) [25] allows for the investigation of relationships between the sensory characterisation completed using the free-sort method and volatile analysis using GC-MS. Due to the use of contingency data for the sensory attributes, means of the four replicates of each sample were calculated for the volatile data, which were unit scaled as part of the MFACT analysis. Only the VOCs determined to be significantly different across the beer samples were included in the MFACT.

All data analysis was completed using R version 3.5.3 [26] and the RStudio IDE [27] with the tidyverse suite of packages [28], plus additional packages agricolae [29], SensoMineR [30], and FactoMineR [31].

## 3. Results and Discussion

### 3.1. Summary of Beer Groups Formed

**Figure 1:**
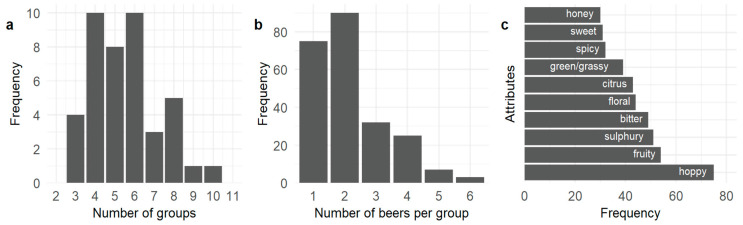
(**a**) Frequency of the number of groups formed by the panelists during the free sorting task (14 panelists × 3 sessions); (**b**) frequency of the number of beers sorted into each group; (**c**) frequency of the ten most commonly used attributes.

In the sorting task, panelists created between three and ten groups from the twelve beer samples (Figure 1a) with four, five, and six groups being most common. The groups most frequently contained two beers (Figure 1b), with one sample per group being the next most frequent, showing that the sensory attributes of the beers were being perceived by the panelists as being distinctly different.

### 3.2. Representation of Beers and Sensory Attributes

A total of 96 distinct attributes were generated by the panelists, which were refined through textual processing to 41. Hoppy was the most commonly used attribute to describe the twelve beers, followed by fruity, sulfury, bitter, floral, citrus, green/grassy, spicy, sweet, and honey (Figure 1c). From the FAST analysis, hoppy was significantly (*p* < 0.05) associated with beer W3470 (Table 2) while sulfury was significantly (*p* < 0.05) associated with samples WY1272 and OTA79. WY1272 was also significantly associated with metallic. WB06 was significantly associated with the terms spicy and estery while WLP730 was significantly associated with spicy. The use of sour was significantly associated with the VIN13 beer sample while astringent was significantly associated with the WLP001 beer. No terms were significantly associated with US05, BE256, S23, OTA29, or SPH.

### 3.3. Representation of Similarity Co-Occurrence Matrix of Beers

The co-occurrence matrix reflects the perceived similarity of the different beers (Table 3). The most similar beers were WB06 and WLP730, associated together 17 times (40.5%); WLP730 and SPH, associated 15 times (35.7%); WB06 and SPH, associated 13 times (31.0%); S23 and WY1272, associated 13 times (31.0%); and US05 was associated with WY1272 and OTA29 12 times each (28.6%). The least similar beers were OTA79 and WLP730, which were not grouped together at all. Samples were grouped alone a total of 75 times, with each sample grouped alone between three and nine times (Table 3). BE256 and VIN13 were most frequently grouped alone nine times (21.4%) while S23 was alone only three times (7.1%), indicating it was perceived as more similar to the other samples.

### 3.4. Relationship of Beer VOCs with Sensory Attributes

**Figure 2:**
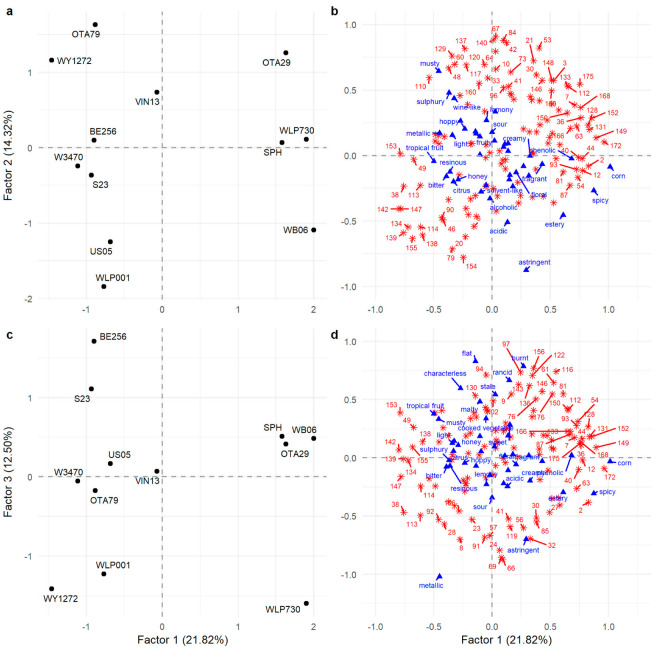
Separation of twelve beer samples (**a**,**c**) with sensory attributes (blue triangles) and VOCs (red asterisks) (**b**,**d**) on the MFACT plot on Factors 1–3. Labelled VOC numbers correspond to important contributors; refer to Appendix A, Table A2 for VOC identification.

The twelve beers were projected similarly in the FAST and MFACT, so to prevent duplication, only the MFACT is presented to illustrate the relationships between the twelve beers and their sensory attributes and VOC profile (Figure 2). Factors 1 (21.82%), 2 (14.32%), and 3 (12.50%) in the MFACT show that the beers were distributed in all dimensions with a total explained variance for these three factors of 48.64%.

Factor 1 separated beers WB06, OTA29, SPH, and WLP730 from the other beers (Figure 2a). This placement was consistent with the co-occurrence similarity matrix where beer WLP730 was grouped 17 times with WB06 and 15 times with SPH, and WB06 and SPH were placed together 13 times (Table 3). The sensory attributes with the highest F1 positive loadings were spicy, corn, phenolic, and estery, while those with the highest F1 negative loadings were bitter, citrus, hoppy, and tropical fruit. This has been reflected in the contingency table (Appendix A, Table A1) where spicy had the highest frequency of use with WB06, WLP730, and SPH; corn and phenolic were associated more frequently with these four beers than any of the others, and estery was most frequently used with WB06. The FAST analysis also reflected this, where spicy was significantly associated with WLP730 and WB06, which was also significantly associated with estery (Table 2). In contrast, citrus and tropical fruit were never used to describe WB06, and although hoppy was used to describe all beers, it was used most frequently with, and was significantly associated to, W3470 while it was used least frequently with WB06.

**Figure 3:**
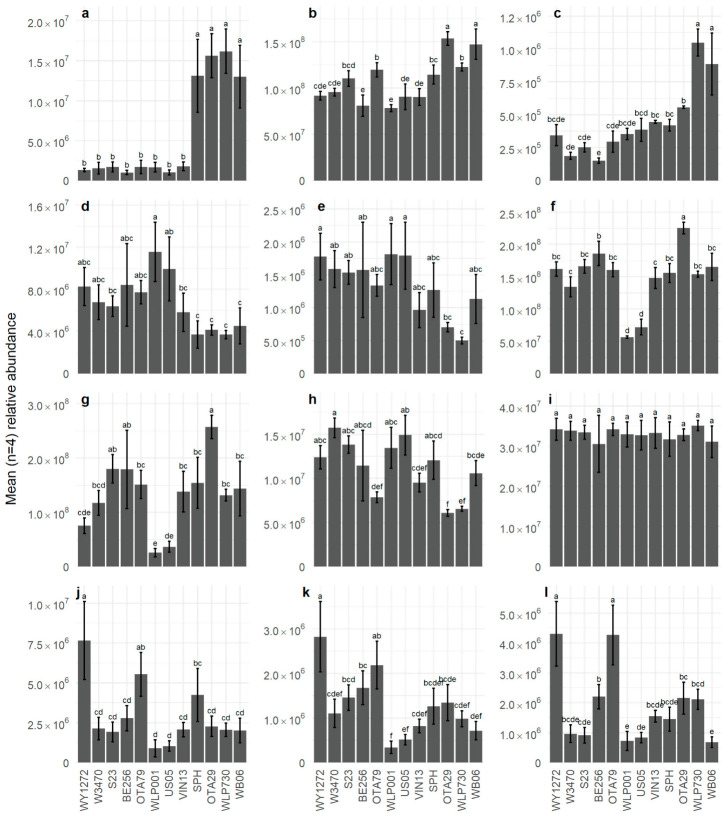
Relative abundance (deconvoluted peak area) of VOC in twelve beer samples. (**a**) 4-vinylguaiacol, (**b**) ethyl hexanoate, (**c**) dimethyl sulfide, (**d**) geraniol, (**e**) nerol, (**f**) isoamyl acetate, (**g**) 2-phenylethyl acetate, (**h**) citronellol, (**i**) linalool, (**j**) citronellol acetate, (**k**) nerol acetate, and (**l**) geranyl acetate. Values are plotted as means ± standard deviation. In each graph, samples sharing the same letter are not significantly different at *p* < 0.05. Note the different y-axis values.

The VOCs with the highest positive loadings on F1 were 4-vinylguaiacol (172; spicy, clove, phenolic), hexanoic acid (149; cheesy), dimethyl sulfide (2; sulfurous, onion, sweet corn, vegetable), 1-propanol (12; alcoholic, earthy, fermented), ethyl hexanoate (36; sweet, fruity, banana, estery), and propyl hexanoate (54; fruity) (Figure 2b). In WLP730, OTA29, SPH, and WB06 beers (Figure 3a), 4-vinylguaiacol was significantly higher. It is produced from malt-derived ferulic acid by heat and/or enzyme decarboxylation, with most conversion (60–90%) attributed to yeast activity. However, not all yeast strains expressed the POF+ (phenolic off-flavour) gene that allowed synthesis of 4-vinylguaiacol from ferulic acid [32]. Hence, the other eight beers only contained trace amounts of 4-vinylguaiacol, as the POF+ gene is not present in the majority of commercial ale and lager yeast strains. The volatile analysis showed that ethyl hexanoate (36) was significantly higher in beers OTA29 and WB06 (Figure 3b). The WB06 beer also showed the highest abundance of propyl hexanoate (54), which corresponds to the literature where WB06 is known to produce estery, fruity, and phenolic flavours [33,34,35]. On F1, there was good agreement between those VOCs with large positive loadings and the sensory attributes used to describe the beers. For example, spicy and phenolic attributes were associated with higher abundance of 4-vinylguaiacol, estery was associated with ethyl hexanoate and other esters, and higher levels of DMS (Figure 3c) were associated with a corn attribute.

The VOCs with the largest negative loadings on F1 were nerol (142; lemon, fruity), geraniol (147; floral, rose, fruity, citrus), ethyl dihydrocinnamate (153; rose, honey, fruity), β-ocimene (38; green, tropical, floral), citral (134; citrus, green, herbal), and citronellol (138; floral, fruity, citrus) (Figure 2b). This reflects the sensory attributes that were also negatively loaded on F1 (bitter, citrus, hoppy, and tropical fruit) and with the citrusy and resinous characteristics consistent with higher levels of the terpene compounds. For example, geraniol (147) was significantly higher in WLP001 and US05 compared to WB06, OTA29, SPH, and WLP730 (Figure 3d), and a similar pattern was found with nerol being lowest in WLP730 and OTA29 (142; Figure 3e).

The separation of beers on F2 was largely due to WLP001, US05, and WB06 with negative loadings and OTA29, OTA79, and WY1272 with positive loadings (Figure 2a). The main drivers in sensory characteristics were the significant association of astringency with WLP001 while OTA79 and WY1271 were significantly associated with sulfury (Table 2). Other negatively loaded sensory attributes included estery, acidic, and alcoholic while hoppy, musty, wine-like, and lemony were positively loaded. The most important positively loaded VOCs on F2 were 2-ethylhexyl acetate (67; earthy, herbal, dirty), octyl acetate (84; green, earthy, citrus), hexyl acetate (42; fruity, green apple), (*E*)-3-hexen-1-ol acetate (53; green, fruity, unripe banana, earthy), geranyl acetate (137; floral, green, citrus, winey), heptyl acetate (64; green, fruity, citrus), nerol acetate (129; floral, fruity, citrus, tropical), 2-phenylethyl acetate (146; floral, rose, honey), and 3-methylbutyl acetate (isoamyl acetate; 21; sweet, fruity, banana). Acetate esters are important flavour compounds in beer and are often present at relatively high concentrations [36]. The presence of these VOCs relates to the positively loaded sensory attributes and relative positioning of the WLP001 and OTA29 beers, which had the lowest and highest abundance of acetate esters, respectively, as illustrated by the abundance isoamyl acetate (Figure 3f) and 2-phenylethyl acetate (Figure 3g).

Factor 3 separated beer BE256 from beers WY1272 and WLP730 (Figure 2c). This separation was due to the sensory attributes metallic and astringent (negative loading) followed by flat, malty, and rancid (positive loadings) (Figure 2d). The FAST analysis identified that metallic was significantly associated with WY1272, which is driving this separation. The VOCs loaded negatively on F3 were 2-methyl-3-heptanone (24; fruity, green, leafy), 4-methyl-2-pentanone (8; solvent, green, fruity, dairy), 2-methylbutyl 2-methylpropanoate (32; fruity, tropical, banana), 2-nonanol (91; waxy, musty, fruity), and 6-methyl-5-hepten-2-one (57; green, musty fruity). In contrast, butyl 9-decenoate (156), 3-methylbutyl octanoate (116; sweet, fruity, green), 2-methylpropyl octanoate (97; fruity, green, floral), and ethyl nonanoate (94; fruity, waxy, tropical) had positive loadings.

### 3.5. Terpenoid Compounds Present in Beers

The primary aim of this study was to evaluate the impact of yeast strain on hop flavour characteristics in beer and to gain an understanding of what VOC in the beers may be responsible for these differences. The relative abundance of the monoterpene alcohols and monoterpene esters in the twelve beers was investigated to see whether any trends existed across the twelve yeast strains.

Hop terpenoids form part of the essential oil component found in hops that contributes aroma-active compounds to beer. Hop terpenoids in beer originate from hops added during the brewing process, which may be modified by biotransformation reactions by yeast during fermentation [5,37]. The monoterpene alcohols geraniol (147), nerol (142), citronellol (138), and linalool (95) are important aroma-active terpenoids found in beer. Geraniol was significantly higher in WLP001 and US05 compared to WB06, OTA29, SPH, and WLP730 beers (Figure 3d). The highest nerol concentration was found in the WLP001, WY1272, and US05 beers, and the lowest concentration occurred in the WLP730 and OTA29 beers (Figure 3e). Nerol contributed a rose-like flavour, and geraniol contributed a rose-like, floral, and citrusy flavour in beer [34]. Although the abundance of geraniol and nerol tended to be highest in the ale *S. cerevisiae* yeast strains, WB06 (*S. cerevisiae* yeast strain) had the fourth lowest abundance of geraniol and nerol, with two *S. pastorianus* strains producing beers with higher levels. King and Dickinson [5] suggested that the extent of monoterpene transformation is strain specific, rather than related to either *S. cerevisiae* or *S. pastorianus* strains.

Citronellol is a yeast biotransformation product of geraniol [5] typically found at low levels in hop essential oil, but at much greater abundance in beer. The abundance of citronellol followed a similar pattern to nerol (Figure 3h). Linalool, one of the most frequently occurring and abundant terpene alcohols in hops, is a product of the oxidation of myrcene, and it is found in hop essential oil. Linalool has been identified as an important odour-active compound in lager beer [38,39,40]. In the current study, the level of linalool (95; Figure 3i) did not significantly differ between the twelve beer samples. This means that although it may have contributed to a generic hop flavour, it did not appear to be an important compound in yeast-specific biotransformations, at least in beers late-hopped with New Zealand Motueka hops.

Citronellol acetate (117) was found at the highest abundance in WY1272, significantly higher than all other beer samples other than OTA79 (Figure 3j), showing a different pattern compared to citronellol. Nerol acetate (129; Figure 3k) and geranyl acetate (137; Figure 3l) illustrated similar patterns, with the highest abundance in WY1272 and OTA79. Citronellol acetate was reported to contribute a floral, fruity, pear, and apple character, and nerol acetate contributed a floral and green flavour to beer [41]. Citronellol acetate is not naturally found in hops, which suggests the production of terpene esters during fermentation by yeast esterase activity. Citronellol acetate is formed either by acetylation of citronellol, after reduction from geraniol, or by reduction from geranyl acetate [42]. It is known that monoterpene acetate esters of terpenes are formed in beer during fermentation and are particularly expressed in high concentration in beers that are late-hopped. King and Dickinson [5] postulated that terpenoid ester formation occurs with lager yeast strains but not in ale strains, although only two strains for each of *S. cerevisiae* and *S. bayanus* were investigated for that study. In contrast, the current experiment, supported by a study conducted by Richter, Eyres, Silcock, and Bremer [3], showed that ale yeast strains can form higher amounts of citronellol acetate, geranyl acetate, and nerol acetate than the selected *S. bayanus* strains. It was also observed that the beers with high levels of the yeast-derived ethyl acetate and higher alcohol acetate esters (e.g., isoamyl acetate, 2-phenylethyl acetate) did not necessarily have the highest levels of terpene acetate esters. This is likely because the formation of the terpene acetate esters requires the presence of abundant monoterpene alcohols, in addition to alcohol acetyltransferase (AATase) activity and acetyl CoA. In contrast, US05 and WLP001, which had amongst the highest levels of geraniol and nerol but the lowest levels of the higher alcohol acetate esters, had the lowest levels of citronellol acetate and nerol acetate. This is probably due to reduced AATase activity rather than reduced acetyl-CoA availability, as yeast growth was not excessive [43].

## 4. Conclusions

The sensory characteristics and VOC profiles of beers fermented by twelve different yeast strains under the same conditions differed considerably, with links being evident between the presence of specific volatile compounds and perceived sensory attributes. Beers high in 4-vinylguaiacol were perceived to be spicy and clove-like, the abundance of dimethyl sulfide was associated with a corn character, and beers with higher concentrations of acetate and ethyl esters were perceived to be fruity and estery. Biotransformation of terpenes involved a complex series of reaction pathways, which led to distinct patterns of hop-derived terpenoid compounds in the twelve beers as a function of yeast strain. The levels of monoterpene alcohols (geraniol, nerol, and citronellol) varied across the twelve samples, although there were no differences in the abundance of linalool. The terpene acetate esters detected varied between the twelve beers, but their occurrence did not correlate with the abundance of fermentation esters or monoterpene alcohols.

To understand the generation of these biotransformation products in beer, further studies need to be conducted under model conditions where the precursors can be closely controlled, to further understand the biosynthetic reactions. Finally, understanding how yeast strain and fermentation factors influence hop aroma in beer will help the brewing industry to better understand how to control beer flavour to meet consumer demands.

## Figures and Tables

**Table 1:** Yeast characteristics and pitching rates.

Yeast Strain ^1^	Supplier	Species	Pitch Rate ^2^	Yeast Type
SafAle US-05 (US05)	Fermentis	*S. cerevisiae*	1 × 10^7^ cells·mL^−1^	Dry
SafAle BE-256 (BE256)	Fermentis	*S. cerevisiae*	9.58 × 10^6^ cells·mL^−1,^*	Dry
SafAle WB-06 (WB06)	Fermentis	*S. cerevisiae*	1 × 10^7^ cells·mL^−1^	Dry
SafLager W-34/70 (W3470)	Fermentis	*S. pastorianus*	1 × 10^7^ cells·mL^−1^	Dry
SafLager S-23 (S23)	Fermentis	*S. pastorianus*	1 × 10^7^ cells·mL^−1^	Dry
VIN13 hybridisedwine yeast (VIN13)	Anchor Oenology	*S. cerevisiae hybrid*	2.50 × 10^6^ cells·mL^−1,^*	Dry
Exotics SPH wine yeast (SPH)	Anchor Oenology	*S. cerevisiae*	5.47 × 10^6^ cells·mL^−1,^*	Dry
Chardonnay white wine yeast WLP730	White Labs	*S. cerevisiae*	1 × 10^7^ cells·mL^−1^	Agar slope
OTA29	University of Otago yeast collection	*S. bayanus*	1 × 10^7^ cells·mL^−1^	Agar slope
California ale WLP001	White Labs	*S. cerevisiae*	4.14 × 10^6^ cells·mL^−1,^*	Liquid
OTA79	University of Otago yeast collection	*S. cerevisiae*	2.54 × 10^6^ cells·mL^−1,^*	Slurry
American ale II strain 1272 (WY1272)	Wyeast	*S. cerevisiae*	3.18 × 10^6^ cells·mL^−1,^*	Slurry

^1^ All fermentations were held at 20 °C. ^2^ Target pitch rate was 1 × 10^7^ cells·mL^−1^. * Under pitched yeast below the target pitch rate.

**Table 2:** Significant association of beer samples to sensory attributes using FAST analysis.

Beer	Descriptor	Intern (%) ^1^	Global (%) ^2^	*p*-Value
W3470	Hoppy	16.67	8.66	0.03
OTA79	Sulfury	14.39	5.89	0.001
WY1272	Sulfury	12.33	5.89	0.04
	Metallic	4.11	0.81	0.03
WLP730	Spicy	11.42	3.70	0.01
WB06	Spicy	10.14	3.70	0.02
	Estery	8.70	2.66	0.01
VIN13	Sour	8.33	3.12	0.04
WLP001	Astringent	3.85	0.81	0.04

^1^ Frequency of use of the descriptor with the sample as a percentage of all descriptors used for the sample. ^2^ Frequency of use of the descriptor across all samples as a percentage of the total number of descriptors used across all samples and evaluations.

**Table 3:** Co-occurrences of beer samples in the sorting task.

Samples	WY 1272	W3470	S23	BE256	OTA79	WLP 001	US05	VIN13	SPH	OTA29	WLP30	WB06	Alone
WY1272		7	13	10	8	6	12	11	3	10	5	2	5
W3470	7		10	6	10	3	11	3	6	8	6	3	5
S23	13	10		11	4	6	7	8	2	7	4	5	3
BE256	10	6	11		5	4	7	8	3	7	3	4	9
OTA79	8	10	4	5		6	6	8	5	10	0	5	8
WLP001	6	3	6	4	6		9	4	11	7	7	5	8
US05	12	11	7	7	6	9		1	5	12	5	4	4
VIN13	11	3	8	8	8	4	1		3	4	7	6	9
SPH	3	6	2	3	5	11	5	3		11	15	13	7
OTA29	10	8	8	7	10	7	12	4	11		7	10	5
WLP730	5	6	4	3	0	7	5	7	15	7		17	4
WB06	2	3	5	4	5	5	4	6	13	10	17		8

## Data Availability

The data presented in this study are available on request from the corresponding author.

## Appendix A

**Table A1:** Frequency of use of distinct descriptors with twelve beer samples.

Attribute	US 05	WLP 001	W 3470	S 23	WB 06	WLP 730	OTA 29	OTA 79	WY 1272	VIN 13	BE 256	SPH	Total
Hoppy	7	6	12	3	2	3	8	11	7	6	6	4	75
Fruity	5	2	6	6	3	5	5	4	8	4	4	2	54
Sulfury	4	1	2	5	3	1	2	12	9	6	4	2	51
Bitter	3	8	7	7	2	3	1	5	5	1	5	2	49
Floral	6	5	2	3	5	3	6	2	3	4	1	4	44
Citrus	5	8	1	6	0	2	2	2	3	6	6	2	43
Green/ Grassy	2	3	4	5	2	2	7	0	6	0	2	6	39
Spicy	1	4	0	1	7	8	2	2	0	1	0	6	32
Sweet	5	2	0	3	5	1	3	2	1	4	3	2	31
Honey	4	3	2	6	2	1	1	1	3	4	2	1	30
Sour	0	2	4	0	3	3	0	2	4	6	1	2	27
Phenolic	2	1	1	2	4	5	4	1	0	2	1	4	27
Woody	2	3	1	2	1	2	4	1	2	2	1	2	23
Estery	3	3	0	1	6	4	2	0	2	0	0	2	23
Malty	1	0	3	4	1	1	1	1	1	0	3	3	19
Lemony	0	0	4	1	1	3	0	1	2	4	1	1	18
Yeasty	2	1	1	0	3	0	1	4	0	2	1	2	17
Solvent-Like	3	2	2	0	2	1	1	2	0	1	2	0	16
Acidic	2	2	3	1	3	2	0	0	1	1	0	1	16
Fragrant	1	1	2	0	2	3	2	1	0	0	2	1	15
Creamy	2	2	0	0	1	1	2	2	1	0	0	3	14
Banana	3	2	0	1	1	0	2	1	1	0	0	3	14
Apple/Pear	1	1	0	2	1	1	1	1	1	3	1	1	14
Caramel	2	2	0	0	0	0	2	2	0	3	2	1	14
Corn	0	1	0	0	3	3	2	0	0	1	1	3	14
Tropical Fruit	1	1	2	3	0	0	1	0	1	2	2	0	13
Light	1	0	1	2	0	2	0	0	2	2	3	0	13
Alcoholic	1	2	3	1	1	1	1	1	0	0	0	1	12
Wine-Like	2	0	1	0	0	0	2	1	2	2	2	0	12
Resinous	0	3	2	1	0	1	1	2	0	0	2	0	12
Flat	0	0	1	3	0	0	1	1	0	0	2	2	10
Grainy	0	1	0	1	1	2	0	0	1	0	2	2	10
Cooked Vegetable	1	1	1	0	1	1	0	1	0	0	3	1	10
Characterless	0	0	1	2	0	0	1	0	1	1	2	1	9
Soapy	1	1	0	1	0	1	1	0	1	2	0	1	9
Rancid	1	0	1	0	1	0	0	0	0	1	2	2	8
Astringent	0	3	0	0	1	2	0	0	0	0	1	0	7
Musty	1	0	0	0	0	0	1	2	1	0	2	0	7
Metallic	1	1	0	0	0	1	0	0	3	1	0	0	7
Stale	0	0	0	0	1	0	0	1	0	1	1	0	4
Burnt	0	0	0	0	1	0	0	0	0	1	1	0	3
Total	76	78	70	73	70	69	70	70	72	74	74	70	866

**Table A2:** Volatile organic compounds identified in twelve beer samples.

No.	Volatile Compound	RI (calc)	RI (lit)	Match	CAS No.	*p*-Value
1	acetaldehyde	713	702	852	75-07-0	0.000
2	dimethyl sulfide	760	754	948	75-18-3	0.000
3	ethyl acetate	896	888	944	141-78-6	0.000
4	ethanol	937	932	935	64-17-5	0.296
5	ethyl propanoate	963	953	793	105-37-3	0.000
6	ethyl 2-methylpropanoate	972	961	780	97-62-1	0.000
7	n-propyl acetate	981	973	942	109-60-4	0.000
8	4-methyl-2-pentanone	1014	1010	891	108-10-1	0.016
9	2-methylpropyl acetate	1019	1012	952	110-19-0	0.000
10	3-methyl-2-pentanone	1025	1019	731	565-61-7	0.000
11	α-pinene	1031	1028	840	80-56-8	0.251
12	1-propanol	1038	1036	938	71-23-8	0.000
13	ethyl butanoate	1042	1035	941	105-54-4	0.000
14	5-methyl-3-hexanone	1082	1036	746	623-56-3	0.334
15	dimethyl disulfide	1083	1077	738	624-92-0	0.000
16	2-methylpropyl propanoate	1085	1079	708	540-42-1	0.002
17	2-methyl-1-propanol	1088	1092	949	78-83-1	0.000
18	2-methylpropyl 2-methylpropanoate	1094	1090	940	97-85-8	0.000
19	1-(1-ethoxyethoxy)-pentane	1107	1098	924	13442-89-2	0.206
20	β-pinene	1115	1112	741	127-91-3	0.020
21	3-methyl-1-butyl acetate	1126	1122	951	123-92-2	0.000
22	ethyl pentanoate	1138	1134	877	539-82-2	0.050
23	5-methyl-2-hexanone	1145	1156	873	110-12-3	0.000
24	2-methyl-3-heptanone	1154	1179	844	13019-20-0	0.001
25	β-myrcene	1167	1161	959	123-35-3	0.079
26	α-phellandrene	1167	1167	863	99-83-2	0.105
27	2-methylpropyl 2-methylbutanoate	1178	1179	827	2445-67-2	0.026
28	2-heptanone	1186	1182	871	110-43-0	0.000
29	α-terpinene	1188	1180	897	99-86-5	0.346
30	2-methylbutyl propanoate	1191	1197	907	2438-20-2	0.000
31	ethyl 4-methylpentanoate	1192	1190	755	25415-67-2	0.000
32	2-methylbutyl 2-methylpropanoate	1197	1199	976	2445-69-4	0.006
33	2-methyl-1-butanol	1201	1208	945	137-32-6	0.000
34	3-methyl-1-butanol	1203	1209	960	123-51-3	0.254
35	β-phellandrene	1218	1211	920	555-10-2	0.940
36	ethyl hexanoate	1236	1233	978	123-66-0	0.000
37	(2R,5R)-2-methyl-5-(prop-1-en-2-yl)-2-vinyltetrahydrofuran	1248	1243	779	54750-70-8	0.164
38	β-ocimene	1255	1250	883	13877-91-3	0.001
39	unknown	1259	-	-	-	0.000
40	styrene	1267	1261	962	100-42-5	0.000
41	4-pentenyl butanoate	1271	-	726	30563-31-6	0.004
42	hexyl acetate	1274	1272	923	142-92-7	0.000
43	bicyclo [4.2.0]oct-1-ene, 7-exo-ethenyl-	1276	-	836	-	0.579
44	ethyl 5-hexenoate	1280	1271	761	-	0.000
45	2-methylbutyl 2-methylbutanoate	1282	1284	935	2445-78-5	0.752
46	ethyl 5-methylhexanoate	1288	-	922	10236-10-9	0.000
47	α-terpinolene	1292	1283	873	586-62-9	0.137
48	1,2,4-trimethyl-benzene	1293	1282	709	95-63-6	0.000
49	ethyl (*Z*)-3-hexenoate	1293	1292	752	64187-83-3	0.000
50	2-methylbutyl 3-methylbutanoate	1297	1299	825	2445-77-4	0.285
51	ethyl (*E*)-3-hexenoate	1302	1289	912	26553-46-8	0.018
52	unknown ester	1316	-	-	-	0.198
53	(*E*)-3-hexenyl acetate	1319	1306	769	3681-82-1	0.000
54	propyl hexanoate	1320	1316	762	626-77-7	0.000
55	ethyl heptanoate	1335	1331	943	106-30-9	0.000
56	methyl 4-methylenehexanoate	1340	1345	817	73805-48-8	0.000
57	6-methyl-5-hepten-2-one	1342	1338	916	110-93-0	0.001
58	1-hexanol	1346	1355	909	111-27-3	0.355
59	ethyl 2-hexenoate	1349	1340	821	1552-67-6	0.000
60	unknown	1351	-	-	-	0.000
61	2-methylpropyl hexanoate	1354	1350	754	105-79-3	0.000
62	rose oxide	1360	1350	895	16409-43-1	0.000
63	3-ethoxy-1-propanol	1373	1373	853	111-35-3	0.000
64	heptyl acetate	1374	1377	905	112-06-1	0.000
65	verbenyl ethyl ether	1376	1377	722	80581-06-2	0.992
66	hop ether	1380	1360	900	344294-72-0	0.032
67	2-ethylhexyl acetate	1384	1420	895	103-09-3	0.000
68	unknown	1387	-	-	-	0.001
69	unknown	1391	-	784	-	0.006
70	2-nonanone	1394	1390	943	821-55-6	0.000
71	2-isobutenyl-4-vinyl-tetrahydrofuran	1400	-	732	-	0.146
72	(*E*)-4-hexenyl butanoate	1404	1478	749	-	0.000
73	1,3-dimethyl-1-cyclohexene	1405	-	787	2808-76-6	0.000
74	2-octanol	1410	1412	777	123-96-6	0.000
75	3-(4-methyl-3-pentenyl)-furan	1425	1429	911	539-52-6	0.225
76	ethyl octanoate	1439	1435	941	106-32-1	0.000
77	1-octen-3-ol	1442	1450	768	3391-86-4	0.976
78	(*Z*)-linalool oxide	1446	1444	800	5989-33-3	0.928
79	1-heptanol	1448	1453	878	111-70-6	0.000
80	6-methyl-5-hepten-2-ol	1455	1465	774	1569-60-4	0.064
81	3-methylbutyl hexanoate	1461	1451	884	2198-61-0	0.000
82	acetic acid	1464	1449	938	64-19-7	0.000
83	3-(2,6,6-trimethyl-1-cyclohexen-1-yl)-2-propenal	1465	-	744	4951-40-0	0.000
84	octyl acetate	1476	1474	903	112-14-1	0.000
85	4-*tert*-pentylcyclohexene	1477	-	717	51874-62-5	0.000
86	nerol oxide	1479	1469	827	1786-08-9	0.556
87	ethyl 7-octenoate	1487	1478	925	35194-38-8	0.000
88	2-decanone	1499	1494	706	693-54-9	0.000
89	unknown	1502	-	704	-	0.001
90	decanal	1505	1498	748	112-31-2	0.000
91	2-nonanol	1510	1521	822	628-99-9	0.000
92	2-acetylfuran	1517	1499	755	1192-62-7	0.008
93	propyl octanoate	1521	1510	935	624-13-5	0.000
94	ethyl nonanoate	1537	1531	914	123-29-5	0.009
95	linalool	1540	1547	975	78-70-6	0.820
96	1-octanol	1550	1557	932	111-87-5	0.000
97	2-methylpropyl octanoate	1553	1548	944	5461-06-3	0.000
98	(*E*)-*p*-2-menthen-1-ol	1567	1571	902	29803-81-4	0.213
99	ethyl 3-nonenoate	1570	-	832	91213-30-8	0.068
100	methyl 4,4-dimethyl-3-oxopentanoate	1577	-	744	-	0.828
101	isopulegol	1578	1571	805	89-79-2	0.669
102	2-methylpropanoic acid	1582	1570	910	79-31-2	0.000
103	fenchol	1589	1582	846	1632-73-1	0.776
104	myrcenol	1603	1585	867	543-39-5	0.758
105	2-undecanone	1604	1598	781	112-12-9	0.000
106	2-decanol	1609	1601	764	1120-06-5	0.016
107	terpinen-4-ol	1611	1602	911	562-74-3	0.953
108	cis-verbenol	1613	1663	781	1845-30-3	0.501
109	caryophyllene	1624	1595	827	87-44-5	0.155
110	citronellol formate	1627	1660	732	105-85-1	0.000
111	(*Z*)-*p*-2-menthen-1-ol	1631	1638	901	29803-82-5	0.440
112	ethyl decanoate	1641	1638	946	110-38-3	0.000
113	2,6-dimethyl-5-hepten-1-ol	1647	1654	730	4234-93-9	0.022
114	4-(1-methylethyl)-cyclohexanol	1649	1667	879	4621-04-9	0.003
115	1-nonanol	1652	1660	875	143-08-8	0.000
116	3-methylbutyl octanoate	1660	1658	920	2035-99-6	0.001
117	citronellol acetate	1663	1660	948	150-84-5	0.000
118	ethyl trans-4-decenoate	1668	1676	891	76649-16-6	0.062
119	ipsdienol	1674	1631	876	35628-00-3	0.025
120	decyl acetate	1681	1680	868	112-17-4	0.000
121	3-methylbutanoic acid	1683	1666	766	503-74-2	0.874
122	ethyl 9-decenoate	1694	1694	917	67233-91-4	0.000
123	humulene	1698	1667	905	6753-98-6	0.411
124	methyl geranate	1703	1686	892	2349-14-6	0.403
125	2-undecanol	1709	1717	861	1653-30-1	0.431
126	endo-borneol	1713	1702	917	507-70-0	0.986
127	3-(methylthio)-1-propanol	1722	1719	903	505-10-2	0.000
128	propyl decanoate	1724	1724	817	30673-60-0	0.000
129	nerol acetate	1728	1724	898	141-12-8	0.000
130	unknown	1734	-	-	-	0.001
131	unknown	1737	-	-	-	0.000
132	2,6-dimethyl-1,5,7-octatrien-3-ol	1740	-	886	29414-56-0	0.067
133	ethyl undecanoate	1741	1739	746	627-90-7	0.003
134	citral	1743	1718	764	5392-40-5	0.000
135	cis-piperitol	1750	1758	780	16721-38-3	0.557
136	1-decanol	1755	1760	879	112-30-1	0.000
137	geranyl acetate	1758	1752	911	105-87-3	0.000
138	citronellol	1760	1765	944	106-22-9	0.000
139	7-methyl-3-methylene-6-octen-1-ol	1784	1800	827	13066-51-8	0.000
140	methyl perillate	1789	-	772	26460-67-3	0.000
141	ethyl 10-undecenoate	1795	-	869	692-86-4	0.000
142	nerol	1798	1797	940	106-25-2	0.000
143	ethyl benzeneacetate	1799	1783	735	101-97-3	0.046
144	myrtenol	1802	1797	753	515-00-4	0.216
145	17-octadecynoic acid	1810	-	792	34450-18-5	0.000
146	2-phenylethyl acetate	1831	1813	787	103-45-7	0.000
147	geraniol	1842	1847	962	106-24-1	0.000
148	ethyl dodecanoate	1844	1841	951	106-33-2	0.000
149	hexanoic acid	1858	1846	977	142-62-1	0.001
150	3-methylbutyl pentadecanoate	1863	1863	827	2306-91-4	0.000
151	benzyl alcohol	1886	1870	754	100-51-6	0.014
152	(*Z*)-ethyl pentadec-9-enoate	1898	-	799	56219-09-1	0.000
153	ethyl dihydrocinnamate	1901	1893	884	2021-28-5	0.000
154	bicyclo [2.1.1]hexane-1-carboxylic acid, 5,5-dimethyl-	1910	-	756	3753-38-6	0.000
155	*p*-menth-1(7)-en-9-ol	1914	1889	836	29548-16-1	0.000
156	butyl 9-decenoate	1918	1874	731	0-00-0	0.001
157	phenylethyl alcohol	1924	1906	944	60-12-8	0.136
158	2-ethyl-hexanoic acid	1961	1960	852	149-57-5	0.923
159	heptanoic acid	1969	1950	722	111-14-8	0.255
160	β-phenylethyl butanoate	1980	1958	785	103-52-6	0.000
161	2-acetylpyrrole	1985	1973	728	1072-83-9	0.484
162	cis-1,3,5-trimethyl-cyclohexane	1996	-	664	1795-27-3	0.473
163	*p*-mentha-1,8-dien-7-ol	2016	2016	756	536-59-4	0.439
164	unknown acid	2028	-	-	-	0.328
165	(*E*)-nerolidol	2034	2042	922	40716-66-3	0.066
166	ethyl tetradecanoate	2048	2049	866	124-06-1	0.036
167	γ-nonalactone	2056	2024	885	104-61-0	0.972
168	octanoic acid	2068	2060	930	124-07-2	0.000
169	nonanoic acid	2183	2171	746	112-05-0	0.000
170	2-phenylethyl hexanoate	2188	2162	716	6290-37-5	0.000
171	epicubebol	2190	2169	752	38230-60-3	0.307
172	4-vinylguaiacol	2214	2188	916	7786-61-0	0.000
173	ethyl hexadecanoate	2253	2251	790	628-97-7	0.062
174	ethyl 9-hexadecenoate	2283	2281	873	54546-22-4	0.218
175	n-decanoic acid	2287	2276	953	334-48-5	0.000
176	9-decenoic acid	2353	2341	931	14436-32-9	0.000
177	neric acid	2363	2366	751	4613-38-1	0.989
